# An Up-to-Date Review of Materials Science Advances in Bone Grafting for Oral and Maxillofacial Pathology

**DOI:** 10.3390/ma17194782

**Published:** 2024-09-28

**Authors:** Carmen-Larisa Nicolae, Diana-Cristina Pîrvulescu, Adelina-Gabriela Niculescu, Dragoș Epistatu, Dan Eduard Mihaiescu, Alexandru Mihai Antohi, Alexandru Mihai Grumezescu, George-Alexandru Croitoru

**Affiliations:** 1 Faculty of Dental Medicine, Carol Davila University of Medicine and Pharmacy, 050474 Bucharest, Romania; carmen-larisa.nicolae@umfcd.ro (C.-L.N.); dragos.epistatu@umfcd.ro (D.E.); alexandru.antohi@drd.umfcd.ro (A.M.A.); alex.croitoru@umfcd.ro (G.-A.C.); 2 Faculty of Chemical Engineering and Biotechnology, National University of Science and Technology Politehnica Bucharest, 011061 Bucharest, Romania; diana.pirvulescu@stud.fim.upb.ro (D.-C.P.); adelina.niculescu@upb.ro (A.-G.N.); dan.mihaiescu@upb.ro (D.E.M.); 3 Research Institute of the University of Bucharest—ICUB, University of Bucharest, 050657 Bucharest, Romania

**Keywords:** bone graft, maxillofacial surgery, nanoparticles, growth factors, bone repair, bone defects, biomaterials

## Abstract

Bone grafting in oral and maxillofacial surgery has evolved significantly due to developments in materials science, offering innovative alternatives for the repair of bone defects. A few grafts are currently used in clinical settings, including autografts, xenografts, and allografts. However, despite their benefits, they have some challenges, such as limited availability, the possibility of disease transmission, and lack of personalization for the defect. Synthetic bone grafts have gained attention since they have the potential to overcome these limitations. Moreover, new technologies like nanotechnology, 3D printing, and 3D bioprinting have allowed the incorporation of molecules or substances within grafts to aid in bone repair. The addition of different moieties, such as growth factors, stem cells, and nanomaterials, has been reported to help mimic the natural bone healing process more closely, promoting faster and more complete regeneration. In this regard, this review explores the currently available bone grafts, the possibility of incorporating substances and molecules into their composition to accelerate and improve bone regeneration, and advanced graft manufacturing techniques. Furthermore, the presented current clinical applications and success stories for novel bone grafts emphasize the future potential of synthetic grafts and biomaterial innovations in improving patient outcomes in oral and maxillofacial surgery.

## 1. Introduction

Bone grafting is an intervention in oral and maxillofacial surgery that is very important in addressing a range of defects and diseases affecting the structural and functional integrity of the jaw and facial bones. This intervention is used in cases of congenital deformities, severe traumas, tumor excision, and illnesses like periodontitis that cause considerable bone loss or deformities. These problems can affect an individual’s life, not only through appearance but also by compromising everyday activities such as chewing, talking, or breathing [1,2].

Periodontal disease is among the leading causes of bone loss in the oral cavity. It is characterized by chronic inflammation and infection of the tooth-supporting structures, which progressively leads to the destruction of alveolar bone. As the bone deteriorates, teeth are no longer well supported, become loose, and may eventually fall out, necessitating reconstructive procedures for bone restoration and dental implant placement [3,4,5].

Jawbone resection is the conventional treatment for benign and malignant tumors in the maxillofacial area. Surgical removal of these tumors often results in significant bone defects that impact appearance and functions such as speech and chewing. Bone grafting is essential for restoring both function and facial symmetry [6,7]. Traumatic injuries to the teeth and jaw, whether from accidents, sports, or prior trauma, can also lead to bone loss that requires grafting. In such cases, bone grafting is used to counteract the effects of bone degradation and promote the formation of new bone in areas that have been injured [8,9,10].

**Figure 1:**
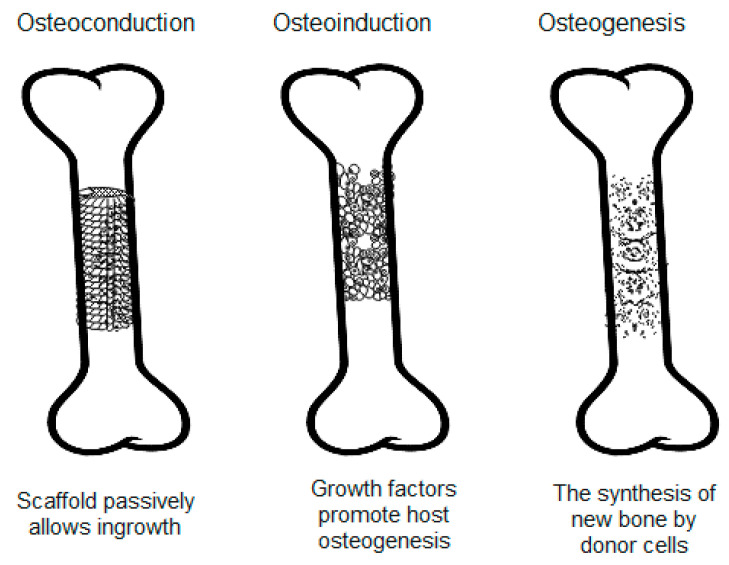
The processes of osteoconduction, osteoinduction, and osteogenesis. Created based on information from [13,14,15].

Bone grafting involves transplanting bone tissue to repair and rebuild damaged bones. This can be done by using the material from the patient′s own body or a synthetic or natural substitute [11,12]. These bone substitutes need to have important biological properties, such as osteoconduction, osteoinduction, and osteogenesis. Osteoinduction is the process by which osteogenesis is induced. Mesenchymal stem cells (MSCs) from a host are recruited to differentiate into chondroblasts and osteoblasts, which form new bone. Osteoconduction involves bone growth on the surface, particularly the growth of capillaries, perivascular tissue, and MSCs. Osteogenesis is the synthesis of new bone from donor cells derived from either the host or graft donor. Cells involved in this process include MSCs, osteoblasts, and osteocytes. These processes are illustrated in Figure 1 [13,14,15].

Traditional bone grafting techniques include autografts, allografts, xenografts, and alloplasts. Autografts are considered the gold standard, involving bone harvesting from the patient’s body. Since the transplant is done from the person’s body, it means it is biocompatible, eliminates the risk of immune rejection, and promotes faster integration and healing. Allografts involve the transfer of grafting materials between two genetically unrelated subjects from the same species, and they have been used as a great alternative to autografts. When autografts and allografts are not practical possibilities, xenografts, derived from a different species (usually bovine and porcine) and alloplasts (synthetic bone substitutes) provide alternatives [13,15,16,17].

Recent advancements in materials science have improved bone grafting procedures. Because autografts, allografts, and xenografts might have limitations, scientists aim to create different bone substitutes using materials and molecules such as bioactive materials, growth factors, nanomaterials, and other tissue engineering approaches [18,19]. Several literature reviews have previously explored the options offered by novel materials for bone regeneration [20,21,22,23,24,25], yet most of them have not been particularized to oral and maxillofacial interventions.

In this context, this paper aims to provide a detailed and up-to-date review of the advances in materials science that are revolutionizing bone grafting techniques in oral and maxillofacial surgery. While most of the previous reviews in this medical area have mainly addressed traditional graft materials such as autografts, allografts, and xenografts [17,26,27,28], this review further identifies and explores the current challenges associated with these options. Moreover, a novel perspective is offered as the main focus of this review comprises cutting-edge developments in materials science, such as combinatorial approaches between growth factors, biologics, and nanomaterials incorporation into better-performing grafts, and advanced manufacturing techniques (e.g., 3D printing and bioprinting). Furthermore, the present paper includes an updated discussion on clinical applications, showcasing successful cases where these new grafting technologies have improved patient outcomes. This approach distinguishes the present review from others in the field, offering an in-depth and updated look at how these advancements reshape the current framework of bone grafting practices in oral and maxillofacial surgery, intending to raise interdisciplinary interest in advancing research and finding even better solutions for the patients in need.

## 2. Autografts, Allografts, and Xenografts and Current Challenges

Autografts involve harvesting bone from the patient’s own body, typically from intraoral and extraoral sites like the iliac crest, cranium, mandible, radius, or tibia, since these are great sources of cortical and cancellous bone [29,30]. Intraoral donor sites for bone transplants have many advantages over extraoral ones, including surgical accessibility, proximity to both the donor and recipient sites, and less pain [31]. Since they come from the patient’s body and possess living cells, they are osteogenic, osteoconductive, and osteoinductive, being the safest biological option since they do not pose a risk to immunogenicity or biocompatibility. However, due to the need for a second surgery, which causes excessive bleeding and scarring, this type of graft might not be suitable for large oral or maxillofacial defects. Furthermore, significant graft resorption is one of the most reported consequences of intraoral harvesting, being linked to substantial morbidity [17,32].

Allografts are extracted from living donors or cadavers, sterilized, and treated to eliminate cells while preserving the bone matrix. As allografts are widely accessible and do not require a separate donor location, they lower patient morbidity. These are usually found as freeze-dried bone, demineralized bone matrix (DBM), or mineralized bone. Allografts have the ability to be osteoconductive and even osteoinductive since they have type I collagen and morphogenetic proteins (BMPs). Most are not osteogenic because they lack living cells [17,33]. Currently, there are some commercially available allografts, for example, Grafton [34], DynaBlast [35], Puros [36], or DBX [37]. However, since allografts are taken from another donor, they have the risk of rejection, disease transmission, and diminished structural integrity due to treatments and irradiation [23,33,38].

Xenografts are derived from non-human sources (bovine or porcine) and offer varying degrees of osteoconductivity. Their main advantage is their wide availability, which makes them a very accessible option. Xenografts’ sources undergo a series of treatments to remove any cellular components that might cause an immune response or transmit a disease [39,40]. Deproteinized bovine bone is the most common source and is currently commercially available as Bio-Oss [41]. Other examples of commercially available xenografts from different sources include OsteoBiol [42], Biocoral [43], Cerabone [44], and Proosteon [45]. While manufacturers of xenografts assure consumers that their goods contain no biological material at all, certain surgeons have found proteins in Bio-Oss following orthognathic surgery [46]. Therefore, the possibility of disease transmission is supported by these findings. Also, the resorption rate tends to be slower than with autografts or allografts, which prolongs the healing process [47,48].

Synthetic bone replacement materials that closely resemble the biological characteristics of natural bone have been proposed to overcome possible immunogenicity and morbidity at donor locations. Their main advantages are the availability, no risk of disease transmission, and the possibility of integrating bioactive molecules into them for faster bone healing. They include materials such as hydroxyapatite (HA), tricalcium phosphate (TCP), bioactive glasses, polymers, and certain metals. However, the only synthetic materials that are currently on the market have osteointegrative and osteoconductive properties [49,50,51]. Some examples of synthetic grafts available on the market are Ostim (contains HA) [52], Osferion (TCP) [53], and Perioglas (bioactive glass) [54].

For an at-glance perspective of the advantages and disadvantages of the above-presented options for bone grafting, Table 1 is included below.

Overall, in spite of their extensive use and benefits, traditional bone grafting techniques still have some disadvantages that affect their efficacy. As a result, there are still challenges in clinical practice [15,58]. Given the limitations of these grafts, there is a clinical need for improved materials and techniques in bone grafting. One of the areas of improvement is represented by the need to encourage the body’s natural ability to regenerate bone, which could be done by creating materials with osteoinductive properties. These should mimic the properties of natural bone, support new bone growth, and integrate with the surrounding tissue, reducing the risk of immune rejection [26,59].

Another important factor to consider is the need for grafting materials to be tailored for each individual in order to match their bone defects and features. Conventional grafts already have pre-defined shapes and sizes, which might not match the bone defect in a patient. These limitations can be overcome using novel technologies such as 3D printing and computer-aided design (CAD), which can produce grafts that precisely match the bone defect [60]. In this manner, essential steps can be taken toward personalized medicine approaches, such as the use of patient-specific implants, that enable accurate and efficient reconstruction of complex maxillofacial defects [61]. Additionally, synergistic strategies corroborating artificial intelligence, computer-assisted surgical planning, and advanced manufacturing methods have the potential to enhance the precision and efficacy of oral and maxillofacial surgical interventions [62].

The biological characteristics of the graft can also be customized. For example, adding stem cells and growth factors may improve healing in individuals with reduced bone regeneration potential [63,64]. Researchers also aim to incorporate bioactive molecules, such as platelet-rich plasma (PRP) and bone morphogenetic proteins (BMPs), into grafting materials. These can be used to mimic the natural processes in the body and allow for faster and more linear bone repair [65]. Nanotechnology is another recent area of interest. Nanoparticles (NPs) may be used for drugs, growth factors, and gene delivery [66]. However, dosage, delivery methods, and any adverse effects must all be carefully considered before using these technologies in a clinical setting. Extensive research is needed to ensure the safe and efficient use of bioactive compounds and NPs in grafting.

## 3. Recent Advancements in Materials Science Applied to Bone Grafting

### 3.1. Growth Factors and Biologics

**Figure 2:**
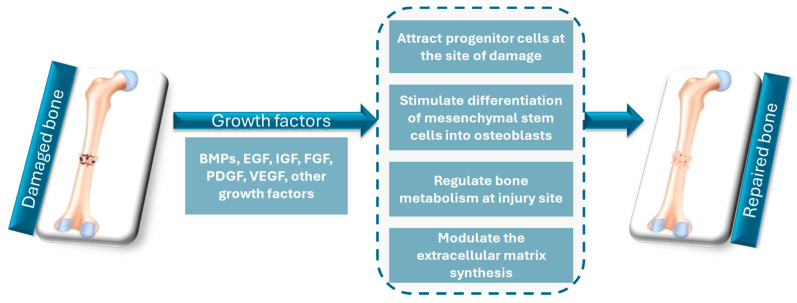
Growth factors involved in bone repair. Created based on information from [70,74,76].

Over the past few decades, much progress has been reported in understanding the molecular and cellular mechanisms of bone healing, leading to the development of growth factors and biologics that increase the effectiveness of bone grafts. Growth factors are essential molecules for tissue repair and regeneration, being involved in cellular proliferation, differentiation, and migration. Specifically, in bone healing, growth factors are engaged in processes that lead to the formation of new bone tissue. Among others, the most well-known growth factors involved in bone regeneration are BMPs, transforming growth factor-beta (TGF-β), insulin-like growth factors (IGFs), platelet-derived growth factors (PDGFs), and vascular endothelial growth factor (VEGF), which are illustrated in Figure 2 [67,68,69,70]. BMPs are the most researched growth factors in bone repair, especially BMP-2 and BMP-7. They are a part of the TGF-β family and possess osteoinductive properties, which allow MSCs to differentiate into osteoblasts (i.e., the cells responsible for bone growth). BMPs trigger a cascade of signaling events: these attract the progenitor cells at the site of damage, promote their growth, and make them differentiate into bone-forming cells. Studies have shown that the use of BMPs can improve bone healing in different applications, such as fracture repair and oral and maxillofacial reconstruction [71,72,73]. TGF-β is another growth factor involved in bone healing. It regulates the proliferation and differentiation of osteoprogenitor cells, modulates the extracellular matrix (ECM) synthesis, and influences the activity of other growth factors involved in bone formation [74]. IGFs, including IGF-1 and IGF-2, are involved in regulating bone metabolism and the anabolic response to bone injury. These growth factors stimulate osteoblast proliferation and differentiation and collagen synthesis, and promote the survival of osteoblasts [75].

Research has investigated the potential of growth factors in bone grafts, showing promising results that could be applied to improve the outcomes of oral and maxillofacial surgeries. For instance, Lee et al. studied a bio-silicated collagen/β-TCP composite as a BMP-2-delivering bone graft substitute for craniofacial bone regeneration [77]. The composite combined the biocompatibility of β-TCP, the structural support of collagen, and the controlled release capabilities of silica NPs (SiNPs). The study found that this composite improved BMP-2 retention and mechanical strength, leading to better bone regeneration in vivo. Wadhwa et al. investigated the effectiveness of different bone graft materials combined with BMP-2 in promoting bone regeneration in rabbit calvarial defects [78]. The researchers compared powdered and block-type tooth-derived autogenous bone grafts with a synthetic bone graft, all combined with BMP-2, and examined the findings after 8 weeks. Both powdered and block-type tooth grafts successfully promoted the formation of new bone by stimulating mesenchymal cells to undergo endochondral ossification. However, the researchers suggest that a longer time than 8 weeks, and better evaluation methods would be necessary to fully observe the osteogenic effects. Another recent study evaluated the effectiveness of recombinant human BMP-2 (rhBMP-2) in promoting bone formation during alveolar cleft reconstruction [79]. It involved 26 patients divided into two groups: one receiving only an iliac crest bone graft (ICBG) and the other receiving ICBG combined with rhBMP-2. It was found that the group treated with rhBMP-2 showed a higher bone formation percentage (55.79%) compared to the ICBG-only group (42.01%). The findings suggest that rhBMP-2 improves bone regeneration in alveolar cleft repair due to its osteoinductive properties. However, despite its promising results, rhBMP-2 does not fully replace autologous bone grafting but rather serves as an adjunct.

One study examined the healing process of soft tissue in the tooth extraction socket (ESsT) after 8 weeks and compared it with subepithelial connective tissue grafts (CTG) typically used in dental surgeries while also observing the roles of TGF-β1 and IGF-1. ESsT showed a strong potential for bone regeneration, as evidenced by high levels of osteogenic markers, unlike CTG. The roles of the two growth factors, TGF-β1 and IGF-1, in bone regeneration were that TGF-β1’s effects vary with concentration: low levels promote bone formation, while high levels inhibit it. IGF-1 generally supports bone formation, but its impact can be suppressed by TGF-β1 [80].

Many other researchers have studied the effect of growth factors in bone grafting for oral and maxillofacial surgery. The studies and their findings are presented in Table 2.

Besides growth factors, biologics, such as stem cells, can also be used in bone grafts. MSCs can be sourced from the patient’s own bone marrow or adipose tissue, minimizing the risk of immune rejection. When delivered to the graft site, these cells can contribute directly to new bone formation and secrete additional growth factors to promote healing [86,87]. A study investigated the use of human adipose tissue-derived MSCs delivered via a Gelfoam^®^ scaffold for repairing maxillary bone defects in rats. In vitro, human MSCs adhered to, proliferated on, and differentiated into bone-forming cells on Gelfoam^®^. The scaffold effectively supported cell growth and osteogenic differentiation. When implanted in rat maxillary bone defects, MSCs on Gelfoam^®^ improved bone regeneration compared to controls with only Gelfoam^®^. Bone formation was much higher and more organized in the MSC-treated groups [88]. Another recent research compared the effectiveness of autogenous bone grafts versus tissue-engineered bone grafts using adipose-derived stem cells (ADSCs) in repairing alveolar cleft defects in dogs. The results showed that both autogenous bone grafts and tissue-engineered bone grafts using ADSCs showed similar bone density measurements over time. The study proved that tissue-engineered bone using ADSCs can be a viable alternative to autogenous bone grafts for large defects, being useful when autograft options are limited due to defect size [89]. One study evaluated the effects of HA/poly-lactic-co-glycolic acid (HA/PLGA) scaffolds implanted with human dental pulp stem cells (DPSCs) for repairing critical-sized bone defects in New Zealand rabbits. HA/PLGA/DPSC scaffolds demonstrated superior bone repair, with more significant radiological improvements and increased radiodensity, especially in the last three postoperative weeks. The addition of DPSCs promoted regeneration, which demonstrated that DPSCs provide osteogenic benefits, which were not achieved with HA/PLGA alone [90].

All these studies suggest that incorporating growth factors and biologicals like stem cells into bone grafts has a great advantage for bone formation and healing. However, more research on extended periods is needed to fully understand their long-term effects.

### 3.2. Nanotechnological Approaches

Nanotechnology is a field used in many research areas, including medicine. It involves using nanoscale materials that exhibit different properties than their bulk counterparts. Their unique chemical–physical characteristics are amplified by their nanoscale size, making them useful in a variety of biomedical applications [91,92]. Based on recent research, NPs can influence bone regeneration by improving osteogenic differentiation, cell proliferation, and signaling [93].

**Figure 3:**
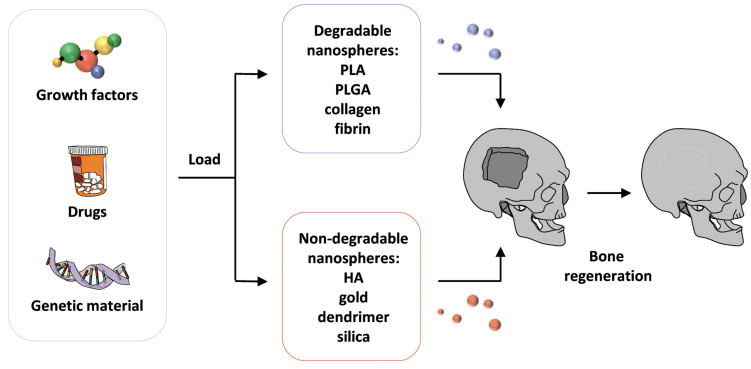
NPs loading for bone regeneration. Reprinted with permission from [66]. Copyright: Elsevier, 2015.

Nanomaterials can be used to incorporate substances, such as growth factors or bioactive molecules, to aid bone regeneration. These molecules can either be incorporated within the nanocarrier or attached to the surface. This allows for a controlled release of the substance directly to the bone defect location, which can improve osteogenesis and the overall effectiveness of the graft [66,94,95]. NPs can be either organic, inorganic, or carbon-based. At the same time, some of them are biodegradable, such as polymers (poly(l-lactide) (PLA), poly(l-lactide-co-glycolic) (PLGA), collagen, fibrin), while others are non-biodegradable (metal NPs, HA, silica) [66,92,96,97]. However, they can all be used in bone grafts for bone regeneration. This process is illustrated in Figure 3.

Polymers are useful in bone grafts since they are biologically similar to bone. They are flexible, lightweight, and can be altered to fit the desired application [98,99,100,101,102]. PLGA has gained interest in bone grafting due to its biocompatibility, biodegradability, and bone regeneration properties [100,103]. A recent study confirmed the benefits of using PLGA in bone grafting, where the researchers developed a nanofibrous collagen/curcumin membrane containing aspirin-loaded PLGA NPs for guided bone regeneration. The prepared membrane exhibited a uniform nanosized fiber network negative surface charge, providing a controlled aspirin release. In a dog model, the membrane was applied to jawbone defects. After 28 days, the test area with the new membrane was completely filled with new bone, whereas the area treated with a commercial membrane remained empty. Even more, a soft tissue layer formed above the formed bone, demonstrating the membrane’s effectiveness in promoting bone and soft tissue regeneration. The study suggests that this asymmetric guided bone regeneration membrane could be used in periodontal surgeries to prevent soft tissue from occupying bone defects while simultaneously promoting bone regeneration [104]. Another study explored the potential of PLGA as coating, and more specifically, they created PLGA-coated vancomycin-loaded silicate porous microspheres for dental bone grafting applications. The ceramic microspheres were made from magnesium–calcium (Mg-Ca) silicates (diopside, bredigite, and akermanite). The uncoated microspheres had a high burst release of vancomycin, while coating the microspheres with PLGA effectively reduced this burst release by improving the diffusion mechanism. The PLGA-coated akermanite microspheres showed the highest cell viability when tested with DPSCs, demonstrating its biocompatibility. These findings suggest that PLGA is a good candidate for creating bone grafts [105].

Collagen (type I) is a major structural component of bone, constituting 90% of the bone ECM’s organic components, making it very useful for bone grafting [106,107]. Its effects have been studied in combination with PLGA in a study by Banche-Niclot et al. [108]. A scaffold based on type I Collagen/PLGA NPs loaded with TGF-β1 was created and investigated for bone defects. Incorporating the NPs into the collagen matrix led to a more sustained release of TGF-β1 than NPs alone. It demonstrated a controlled release of TGF-β1, reducing the initial burst release from 38% to 14.5% within the first 24 h. The study is significant as it is the first to report the design of collagen-based scaffolds embedding PLGA NPs for TGF-β1 release similar to natural human bone ECM. This research suggests that such composite scaffolds could be very useful in bone regenerative therapies by providing controlled delivery of growth factors.

HA is widely used in bone tissue engineering due to its bioactivity and biocompatibility and because it is one of the primary components in bones [109,110]. Since HA has osteoconductive and osteoinductive properties, osteoblastic cells adhere to and proliferate on the surface, facilitating the attachment between implant and bone tissue. However, due to its poor mechanical properties, HA is usually used with other bone regeneration materials [111,112,113,114]. Calabrese et al. focused on Mg-HA-Coll type I scaffolds, which they functionalized with gold nanorods (Au NRs), palladium NPs (Pd NPs), and maghemite NPs (MAG NPs) to assess their impact on the proliferation and differentiation of human adipose-derived MSCs (hADSCs) for bone regeneration [115]. Scaffolds functionalized with MAG NPs significantly improved cell proliferation by 70% compared to the control scaffold and showed a 25% increase in calcium deposits, a key indicator of bone formation, compared to the control. This suggests that MAG NPs promoted cell growth and supported the scaffold’s ability for bone differentiation. The incorporation of HA in the scaffold ensured its osteoinductive properties, leading to increased calcium deposition and bone differentiation and providing structural integrity. Demirel et al. evaluated the effects of doping nano-gold (nAu) and nano-silver (nAg) into HA-based bioceramic bone grafts. Cell viability tests using human fibroblast cells showed that pure HA and HA with a low concentration of nAu (HA-nAu5) were non-toxic at all tested concentrations. However, at higher concentrations, most nAu- and nAg-doped grafts displayed toxic effects. The use of HA ensured the graft’s biocompatibility and osteoinductive properties. However, while nAu and nAg can enhance certain properties of HA-based bone grafts, they also introduce cytotoxicity, particularly at higher concentrations. Therefore, more research is needed to fully understand the right concentrations when combining HA with other materials with potential toxicity.

Many other NPs have been studied over the years for their potential benefits in bone grafting. A few of these findings are summarized in Table 3.

These studies highlight the benefits of nanomaterials’ benefits for bone regeneration and healing; however, their effects depend on the materials, concentrations, and formulations used. When developing NPs to be incorporated within synthetic bone grafts, it is paramount to investigate their safety. A critical issue is the potential toxicity of NPs, which can result from their small size, high surface area, and ability to penetrate biological barriers. These factors can lead to unintended interactions with cells and tissues, triggering inflammation, oxidative stress, and cellular damage [122,123]. To mitigate these risks, rigorous preclinical studies are essential to assess the biocompatibility, degradation, and clearance of NPs from the body. Ethical considerations also demand transparency in the research and development processes.

Thus, more research is needed to thoroughly evaluate the long-term effects and potential negative effects of NPs in these applications. Addressing these risks through comprehensive research, transparent patient communication, and responsible use is essential to ensuring that the benefits of nanotechnology in bone grafting outweigh its potential harms.

### 3.3. Advanced Manufacturing Techniques

The evolution of manufacturing techniques has significantly impacted the development of bone grafting materials for oral and maxillofacial surgery. Depending on the type of graft, different materials are used for its fabrication. The materials’ classification is illustrated in Table 4 [28].

Currently, several methods of graft fabrication are available. Some examples of conventional graft fabrication methods include freeze-drying, solvent casting, and sintering of ceramic powders. However, these have their limitations, such as lack of precision, making personalization difficult [124,125]. Therefore, new advancements in fabrication techniques have been made to overcome these limitations, which will be further discussed.

#### 3.3.1. Additive Manufacturing and 3D Printing Techniques

Oral and facial bone repair is a very complex process due to the anatomical individuality of each lesion, the possibility of infection, etc. Available treatments for large bone defects are limited due to the complexity of bone defects and their discrepancies across individuals. 3D printing is a subset of additive manufacturing (AM), which involves creating a 3D structure based on a computer model. Personalized bone grafts can be printed using 3D printing as it could enable the creation of bone substitute materials with tailored form, porosity, and topography. This can be done by using imaging techniques such as computed tomography (CT) or magnetic resonance imaging (MRI) to make detailed 3D models of the patient’s anatomy [50,63,126,127].

Recent research involved creating innovative approaches to 3D printing bone grafts by combining extrusion deposition techniques with new emulsion inks to enhance scaffold properties [128]. Propylene fumarate dimethacrylate (PFDMA) was chosen for fabricating the bone grafts due to its established biocompatibility, osteoconductivity, and good compressive strength. Scaffolds made from PFDMA had a compressive modulus of 15 MPa and yield strength of 1 MPa. Lowering the scaffold infill increased permeability but reduced strength. To balance strength and permeability, scaffolds were reinforced with an outer shell of thermoplastic polyester (poly(ε-caprolactone) (PCL) or PLA). PLA-reinforced scaffolds had a higher compressive modulus and yield strength than PCL-reinforced scaffolds. The study developed a multi-modal printing system that combined paste extrusion (for the emulsion inks) and high-temperature thermoplastic extrusion (for the PCL or PLA shells), which allowed for high positional accuracy and dual deposition, making scaffolds with better mechanical properties.

Anbu et al. have evaluated three types of materials for bone grafting in vivo: Ossifi bone graft (Equinox), powdered PLA, and 3D-printed PLA [129]. After 4 weeks, PLA materials (both powdered and 3D printed) showed less bone formation compared to Ossifi. However, 3D-printed PLA demonstrated early signs of bone regeneration with apposition around the scaffold, suggesting good integration. It also demonstrated precision in matching defect geometry. This study suggests that 3D-printed PLA is a great alternative to traditional graft materials because it has benefits in scaffold design and bone regeneration. Korn et al. investigated a 3D-printed, biodegradable calcium phosphate-based bone graft for repairing alveolar clefts in a rat model [130]. The 3D-printed scaffolds fit well into the alveolar defects and supported bone regeneration. After 12 weeks, the scaffolds demonstrated effective osteoconduction, with scaffold A (60° pore orientation) showing significantly better results than scaffold B (30° pore orientation). Another interesting observation was that colonizing scaffolds with rat mesenchymal stromal cells (rMSCs) did not make a difference in bone formation compared to non-colonized scaffolds. This suggests that for this particular application, adding cells may not provide additional benefits in terms of defect healing. Saed et al. studied the use of 3D printing to create Poly L-lactic acid (PLLA) scaffolds with added biphasic calcium phosphate (BCP) particles for bone grafting [131]. The scaffolds were fabricated using digital light processing (DLP) 3D printing. This method allowed for precise control over the scaffold’s geometry and porosity. The study identified 22.5% BCP as the optimal concentration for maintaining good printability while enhancing the scaffold’s bioactivity. Scaffolds with this concentration showed improved cell adherence and slower degradation rates. The technique allowed for precise customization of scaffold properties. However, careful consideration of BCP concentration is needed to balance mechanical strength and bioactivity.

#### 3.3.2. 3D Bioprinting

3D bioprinting is a form of 3D printing focusing more on creating structures resembling human tissues. The process involves the layer-by-layer deposition of bioinks, which are materials composed of living cells, growth factors, and biomaterials, to create tissue structures and organs [132,133]. This process has been researched due to its huge potential to aid in bone regeneration because of the bioinks used. A recent study conducted by Amler et al. examined how different sources of human mesenchymal progenitor cells (MPCs) from various bone types perform in 3D bioprinted bone grafts, which were based on methacrylated gelatin (GelMA) [134]. Using projection-based stereolithography, the researchers bioprinted bone tissue constructs incorporating MPCs from alveolar bone, iliac crest, fibula, and periosteal bone shaving. Over a 28-day cultivation period, the constructs were evaluated for cell viability, mineralization, and gene expression related to bone formation. All cell types maintained comparable viability, but periosteal-derived MPCs showed superior bone mineralization and osteogenic differentiation compared to other sources. Periosteal cells also offered advantages due to their minimally invasive harvesting and faster expansion. The study demonstrated that GelMA-based bioink facilitated good cell adhesion and structural integrity. Another study investigated the performance of four different alginate-based bioinks: alginate–calcium chloride (alg-CaCl_2_), alginate–calcium sulfate (alg-CaSO_4_), alginate–gelatin (alg-gel), and alginate–nanocellulose (alg-ncel) for 3D bioprinting osteogenic tissue grafts [135]. The findings revealed that bioinks containing alg-CaSO_4_ and alg-ncel showed better mechanical strength and resolution but led to higher cell apoptosis and fewer living cells compared to the other two. Constructs made with alg-CaCl_2_ demonstrated superior cell–matrix interactions and better osteogenic differentiation of MSCs when cultured in osteogenic media. These constructs were also partially mineralized after 14 days, concluding that Alg-CaCl_2_ was the most effective bioink. Dubey et al. investigated a novel bioink, ECM/AMP, a hydrogel based on a synthetic ECM peptide with amorphous magnesium phosphate (AMP) particles [136]. Bioprinted constructs with the ECM/AMP bioink showed better mineralization and osteogenic gene expression compared to those with the ECM-only bioink. In vivo tests demonstrated that constructs made with ECM/AMP created greater bone volume and density over time compared to ECM-only constructs.

Overall, research indicates that using both 3D printing and 3D bioprinting has benefits because it allows for the creation of personalized grafts that better fit bone defects. Even more, 3D bioprinting is considered a better option in certain applications. Because of bioink use, certain bone regeneration processes are improved, leading to faster healing.

Nonetheless, certain challenges remain despite the transformative potential of 3D printing and bioprinting for bone graft fabrication. Associated costs, accessibility, and technical issues need to be addressed to make these technologies more widely available. In more detail, the high-performing printers required for top-quality biocompatible bone grafts, especially those involving bioinks, growth factors, or stem cells, come with substantial upfront and maintenance costs, posing a financial barrier in their broad implementation [137,138,139]. Hence, in lower-income regions or smaller clinics, the adoption of 3D-printed bone grafts may be financially unfeasible, potentially exacerbating disparities in healthcare access. Therefore, future considerations should also be directed to ensuring the cost-effectiveness of proposed alternative solutions.

## 4. Clinical Applications

The field of bone grafting in oral and maxillofacial surgery has evolved considerably with advancements in materials science. These developments led to various clinical applications and success stories that highlight the impact of material innovations on bone grafting. Examples of these clinical applications are presented in Table 5.

These clinical trials provide information about how different types of bone grafts perform in real-life applications on patients. It has been found that certain types of synthetic grafts performed similarly to autografts, which is a very important finding since autografts may not always be available for some patients. Considering the studies presented, no significant adverse events were reported besides the ones that are considered expected (like pain and swelling), which also happen when using autografts or xenografts.

Besides tabulated studies, several clinical trials have been recently conducted or are ongoing, as identified on the ClinicalTrials.gov platform. The combined search of “oral surgery” and “bone grafting” retrieved 39 results comprising 20 “Completed” studies (out of which five have publicly available results), two “Terminated” clinical trials, two “Active, not recruiting” studies, and seven studies still in early stages (five “Recruiting” and two “Not yet recruiting”) [148]. Therefore, future perspectives can also emerge from the findings of these studies, especially once all the results are published and correlated. However, the number of clinical studies is quite small compared to other medical fields, denoting a slow translation of newly developed solutions to the clinical setting.

This highlights the importance of extending pre-clinical studies on novel graft materials that are still being researched, as they could provide benefits in human trials and potentially lead to an alternative treatment for bone defects.

## 5. Conclusions

Integrating materials science with bone grafting techniques in oral and maxillofacial surgery has led to new discoveries for bone graft creation, which could act as alternatives for current treatments, offering new solutions to the complex challenges of bone regeneration and defect repair. Autografts remain the gold standard in oral and maxillofacial surgical interventions due to their osteogenic, osteoinductive, and osteoconductive properties, lack of immune rejection risk, and no disease transmission threat. However, limitations such as donor site morbidity and limited availability have driven the development of alternative materials. Allografts and xenografts provide good alternatives with good clinical outcomes. However, there are still concerns about immune response and disease transmission. Synthetic bone substitutes have been created as viable alternatives to conventional grafts. They offer several important advantages, such as unlimited availability and the possibility of manufacturing them to exactly match the bone defect, allowing for a personalized treatment.

A critical advancement in improving bone grafting materials is the incorporation of various moieties into the graft. Growth factors, stem cells, and NPs are just a few examples of substances that can be beneficial for bone repair. These innovations aim to mimic the natural bone healing process more closely, promoting faster and more complete regeneration. Manufacturing processes, like 3D bioprinting, can bring these combinations of materials (usually referred to as bioinks) to life. This manufacturing process also allows the creation of a graft that can closely match the bone defect.

Despite these advances, several challenges remain in bone grafting for oral and maxillofacial surgery. The mechanical properties of synthetic grafts often fall short of those of natural bone. The biocompatibility and biodegradability of these materials must be carefully balanced to ensure that they support bone regeneration without causing negative immune responses. Moreover, the long-term stability of grafts, particularly those combined with other molecules, requires further investigation. Their long-term effects are still not fully understood.

As there is no single grafting material or technique that is universally superior, the future of successful bone grafting interventions relies on principles of personalized medicine. The choice of graft depends on a range of factors, including the size and location of the defect, the patient’s overall health, and available options for each case. Thus, particularized approaches are to be expected to improve the outcomes of each and every individual needing oral or maxillofacial surgery.

Therefore, with more research, there is a possibility of developing new bone grafts using biocompatible materials in combination with substances or molecules that promote bone growth and repair while also integrating advanced manufacturing technologies, providing new alternatives to current treatments.

## Figures and Tables

**Table 1:** Comparison of different bone graft options.

Type of Graft	Source	Advantages	Disadvantages	Refs.
Autograft	Bone from the patient’s own body (e.g., from intraoral sites, iliac crest, cranium, mandible, radius, or tibia)	Osteogenic Osteoinductive Osteoconductive Biocompatible Very low risk of immune rejection No disease transmission Fast bone regeneration	Risk of infection Possibility of insufficient bone for transplant Need for extra surgery, which might result in pain, long recovery time, scars Donor site morbidity	[18,26,55]
Allografts	Freeze-dried bone, demineralized bone matrix (DBM), or mineralized bone extracted from living donors or cadavers	Osteoinductive Osteoconductive Single surgery needed Ease of harvesting	Risk of disease transmission Risk of immune rejection Blood incompatibility Diminished structural integrity due to irradiation	[13,18,26,56]
Xenografts	Deproteinized bone material extracted from non-human sources (bovine or porcine)	Osteoconductive Availability Similarity with human bone Low cost	Possibility of disease transmission Immunogenicity	[18,26,57]
Synthetic bone substitutes	HA, TCP, bioactive glasses, polymers, and some metals	Osteoconductive Availability Other advantages depend on the materials	Disadvantages depend on the materials	[16,26]

**Table 2:** Summary of recent studies using growth factors in bone grafts.

Graft	Targeted Defect	Findings	Refs.
Autogenous and xenogenous bone grafts with platelet-rich fibrin (PRF)	Vertical and horizontal bone augmentation prior to dental implant placement	Achieved average bone gains of 5.9 mm in thickness and 5.6 mm in height Improved graft handling and stability PRF enhanced soft tissue healing	[81]
Concentrated growth factors (CGFs) with deproteinized bovine bone mineral (DBBM)	Alveolar ridge preservation (ARP) following upper molar extraction	Bone area in the CGFs/DBBM group was better maintained compared to the control CGFs promoted better osteogenesis and bone integration CGFs as a membrane alternative to collagen—biocompatible; promoted soft and hard tissue growth without additional trauma	[82]
Leukocyte and PRF (L-PRF) with autogenous bone graft and the other Enamel Matrix Derivative (EMD) with autogenous bone graft	Intrabony defects (IBDs)	Both treatments were effective clinically and radiographically in promoting periodontal regeneration Stimulated cellular activities for bone regeneration and periodontal healing	[83]
Mineralized collagen loaded with BMP-2 and VEGF	Bone regeneration in mandibular defects	The combination of BMP-2 and VEGF resulted in the highest level of bone regeneration BMP-2 promoted osteogenesis by stimulating MSCs to become osteoblasts VEGF promoted angiogenesis BMP-2 and VEGF were more effective than using BMP-2 alone	[84]
Autologous platelet concentrates: PRF, CGF, and platelet-poor plasma (PPP)	Healing of tooth extraction sockets	All three platelet concentrates (PRF, CGF, PPP) improved healing and reduced bone resorption compared to control PPP showed the most significant early-stage bone healing effects CGF demonstrated superior results in later stages of healing RF was less effective compared to PPP and CGF Larger areas of bone formation were observed, the most significant being CGF	[85]

**Table 3:** Study-based findings of NPs in bone grafting.

NPs/Graft	Application	Findings	Refs.
Bovine bone grafts with AgNPs	Dental bone grafts	Strong antimicrobial effects Potential of reducing infections in maxillofacial surgeries	[116]
Chitosan NPs (ChN)/Simvastatin (Sim)	Maxillary bony defect healing	ChN had the highest number of osteoblasts and osteoclasts Combining ChN with Sim did not enhance bone healing ChN’s effectiveness may be due to its ability to control drug release and its biocompatibility	[117]
Titanium dioxide NPs (TiO_2_NPs)/TiO_2_NPs-PRP	Healing large bone defects	TiO_2_NPs improved bone healing by promoting osteoblast functions PRP provides essential growth factors that promote vascularization and soft tissue healing TiO_2_NPs-PRP showed the most significant improvement in bone healing PRP enhanced early blood clot formation and sustained angiogenesis, while TiO_2_NPs provided a conducive environment for bone tissue development	[118]
Dental nano putty (D-nP) combining DBM, calcium sulfate hemihydrate (CSH), curcumin NPs (CU-NPs), AgNPs	Bone regeneration and prevent implant-associated infections	D-nP demonstrated effective antibacterial activity against both Gram-positive and Gram-negative bacteria D-nP had high cell viability (~95%) and promoted cellular adhesion and proliferation, which is important for bone regeneration The combination of DBM, CSH, CU-NPs, and AgNPs resulted in a material with improved mechanical strength	[119]
Cerium oxide (CeO_2_) NPs loaded nanofibrous membranes	Periodontal tissue repair	CeO_2_ NPs were biocompatible and promoted the proliferation of human periodontal ligament stem cells (hPDLSCs) Increased alkaline phosphatase activity, mineralized nodule formation, and the expression of osteogenic genes and proteins In rat cranial defect models, the composite membranes with CeO_2_ NPs accelerated new bone formation	[120]
Nanocomposites of Chitosan/Graphene Oxide/TiO_2_ NPs/Blackberry Waste Extract	Partial bone substitutes	Demonstrated good biocompatibility Soft tissue healing, hair regrowth, and no necrotic or inflammatory responses after 90 days of implantation in skull defects Promoted bone cell adhesion and proliferation	[121]

**Table 4:** Bone grafting materials classification. Created based on information from an open-access source [28].

Bone Grafting Material	Description	Examples
Autogenous bone	Bone from the same individual	Block graft
Allogenic bone	Bone from the same species but from a different individual	Free frozen bone, freeze-dried bone allograft, demineralized freeze-dried bone allograft, and deproteinized bone allograft
Xenogenic bone	Material of biological origin but from a different species	Materials derived from animal bone, corals, calcifying algae, and wood
Alloplastic bone	Material of synthetic origin	Calcium phosphates, glass ceramics, polymers, and metals

**Table 5:** Summarization of clinical applications for bone grafts.

Clinical Study Graft	Targeted Defect	Clinical Study Details	Results	Refs.
CAD/CAM titanium meshes filled with graft material (from patient and bovine source)	Guided bone regeneration of severe alveolar ridge defects	41 patients enrolled between 2018 and 2019 The mean duration of mesh maintenance was 7 months	Eight of these sites integrated the graft uneventfully, and three showed partial bone loss 100% survival rate of implants after a follow-up of 10.6 months	[140]
Autogenous dentin as a graft	Periodontal defects caused by the extraction of impacted lower third molars	15 patients recruited and selected over a period of 12 months Evaluations performed at 3 and 6 months post-operatively	The dentin graft showed significant improvements in probing depth (PD), bone density, and maintenance of the alveolar bone crest compared to the control Reduced periodontal pocket depth and improved bone healing	[141]
Octacalcium phosphate (OCP) and its collagen composite (OCP/Col) as bone substitutes	Oral and maxillofacial surgeries: sinus floor elevation, socket preservation, cyst removal, and alveolar cleft repair	60 patients Abutments were exchanged, and prosthetic treatment started 6 months after implant placement	OCP/Col was successful in sinus floor elevation (1-stage), cyst, and alveolar cleft procedures, meeting the criteria for success OCP/Col was deemed safe, with adverse events like pain and swelling being typical of normal treatment	[142]
Synthetic graft volume-stable collagen matrix (VCMX) versus autogenous subepithelial connective tissue graft (SCTG)	Grafting around dental implants	20 patients Re-examinations at 6 months, 1 year, and 3 years	Both VCMX and SCTG led to slight increases in buccal mucosal thickness over three years Initially, patient morbidity was higher with SCTG, but over time, satisfaction levels for both groups were high Both VCMX and SCTG provided stable, healthy, and aesthetically pleasing outcomes, with VCMX offering the advantage of reduced patient morbidity	[143]
Novel 3D-printed nano-porous HA (3DP HA) versus nano-crystalline bone graft (NanoBone^®^)	Preserving the alveolar ridge after tooth extraction	30 patients Evaluation at 4 months after implant placement	After four months, both groups showed similar outcomes in terms of bone resorption, soft tissue changes, and new bone formation Both materials effectively minimized ridge resorption and provided stable conditions for implant placement	[144]
AM CaOSiO_2_-P_2_O_5_-B_2_O_3_ glass-ceramic (BGS-7) implants	Reconstructing zygomatic bone defects	8 patients Follow-up study performed after 4 years	CT scans showed 100% bone fusion at 6 months The mean displacement of implants was minimal, indicating good stability Patients reported high satisfaction with both aesthetic and functional outcomes No adverse effects were reported	[145]
Allograft versus TCP/HA bone substitute	Benign cavitary bone lesions	15 patients included in the study between 2016 and 2019 Follow up every 3 weeks until 6 months, and then at 2-month intervals until one year	Both treatments resulted in similar healing times, with all lesions disappearing after 12 months Both types of grafts showed good integration and similar clinical outcomes The TCP/HA graft is a cost-effective alternative but is more fragile	[146]
5 grafts: (1) Autogenous graft (2) β-TCP graft (3) β-TCP + Autogenous Bone Graft (4) Bioactive Glass (5) Bioactive Glass + Autogenous Bone Graft	Reconstructing maxillary sinuses	40 patients Evaluation performed after 6 months of bone healing	Bioactive glass + Autogenous bone graft outperformed other combinations with 45.8% new bone formation and 37.9% bone volume change Bone graft resorption rates were similar across the different graft materials Combination grafts generally had better outcomes compared to single-material grafts, but were similar to autogenous graft	[147]
